# Feasibility and pilot study of a brief self-compassion intervention addressing body image distress in breast cancer survivors

**DOI:** 10.1080/21642850.2021.1929236

**Published:** 2021-05-21

**Authors:** Angela Mifsud, Melissa J. Pehlivan, Paul Fam, Maddison O’Grady, Annamiek van Steensel, Elisabeth Elder, Jenny Gilchrist, Kerry A. Sherman

**Affiliations:** a Centre for Emotional Health, Department of Psychology, Macquarie University, Sydney, Australia; b Westmead Breast Cancer Institute, Westmead Hospital, Sydney, Australia; c Macquarie University Hospital, Sydney, Australia

**Keywords:** Breast cancer, self-compassion, body image, e-health intervention, feasibility

## Abstract

**Background:**

The majority of breast cancer survivors (BCSs) experience body image concerns following treatment. Body Image distress (BID) is associated with psychological distress and diminished quality of life. A web-based self-compassion focused writing activity (My Changed Body – MyCB) reduces BID in BCSs, yet limited research exists on participant characteristics associated with such intervention adherence. Self-compassion-based meditations are also efficacious in reducing BID in non-BCS populations. This parallel, double-blind pilot randomised controlled trial aimed to assess the feasibility and acceptability of MyCB, with and without an additional meditation component, on BID and related psychological outcomes in BCSs. The trial was registered with the Australian and New Zealand Clinical Trials Registry (#ACTRN12619001693112).

**Methods:**

BCSs were randomly allocated to MyCB (*n* = 39), MyCB + Meditation (MyCB + M) (*n* = 17) or an expressive writing (EW) active control arm (*n* = 23). The primary outcome was BID. Secondary outcomes were body appreciation, affect (positive and negative), psychological distress (depression, anxiety and stress) and self-compassion (state and trait). Assessments were completed online at baseline, post-intervention and 1-month.

**Results:**

Adherence to the MyCB writing (45%) and meditation (50%) was modest, and acceptability was high for both MyCB and MyCB + M. Intent to treat linear mixed model analyses indicated: Post-intervention – state self-compassion and positive affect increased for MyCB compared to EW; 1-month: BID scores decreased across all conditions; trait self-compassion increased and anxiety decreased for MyCB + M compared to MyCB and EW.

**Conclusion:**

These findings provide preliminary evidence for the efficacy and potential clinical use of the MyCB brief web-based self-compassion intervention alone and with the addition of meditation, to increase self-compassion and psychological wellbeing in BCSs.

## Introduction

Breast cancer is the most commonly diagnosed cancer among females worldwide (Bray et al., 2018). Recent improvements in health care, including earlier detection and effective treatment strategies, have significantly improved survival rates (American Cancer Society, 2020; Australian Institute of Health and Welfare, 2019; Dafni, Tsourti, & Alatsathianos, 2019). Unfortunately, breast cancer treatments can result in various adverse effects, including restricted movement, fatigue (Binkley et al., 2012), hair loss, weight fluctuation, breast disfigurement (Helms, O’Hea, & Corso, 2008), sexual problems (Alicikus et al., 2009), pain, infertility (Ewertz & Jensen, 2011), nausea and scarring (Runowicz et al., 2016). Understandably, these changes can lead to distress in many individuals, with the majority of breast cancer survivors (BCSs) experiencing concerns related to their body image (Begovic-Juhant, Chmielewski, Iwuagwu, & Chapman, 2012; Kocan & Gursoy, 2016). Body image refers to subjective perceptions, thoughts, and feelings about one’s entire body in the context of the relative importance placed on physical appearance and body integrity (Fingeret & Teo, 2018). More than half of BCSs report poor body image (Ussher, Perz, & Gilbert, 2012), with at least a third experiencing ongoing body image-related distress years after recovery (Falk Dahl, Reinertsen, Nesvold, Fosså, & Dahl, 2010; Kang et al., 2018).

Body image distress (BID) in BCSs is associated with impairments in work, social and sexual functioning (Boquiren et al., 2016; Fobair et al., 2006; Ljungman et al., 2018)**,** reduced sense of femininity and attractiveness (Kocan & Gursoy, 2016), poorer physical health (Moreira & Canavarro, 2010) and quality of life (Begovic-Juhant et al., 2012; Falk Dahl et al., 2010; Paterson, Lengacher, Donovan, Kip, & Tofthagen, 2016), and higher levels of psychological distress (Chen, Liao, Chen, Chan, & Chen, 2012; Galiano-Castillo et al., 2014; Rosenberg et al., 2013). For many BCSs, these body-image related concerns have not been adequately addressed by health care professionals (Jørgensen, Garne, Søgaard, & Laursen, 2015), whilst interventions targeting BID in BCSs have demonstrated limited efficacy (Lewis-Smith, Diedrichs, Rumsey, & Harcourt, 2018).

An emerging approach to managing body image concerns is the application of self-compassion – the awareness and understanding of one’s suffering, viewing it as part of the human experience and being kind to oneself (Neff, 2003, 2016a). Greater self-compassion is associated with fewer body image concerns, more positive body image (Przezdziecki et al., 2013; Turk & Waller, 2020; Wasylkiw, MacKinnon, & MacLellan, 2012), and less psychological distress (MacBeth & Gumley, 2012; Wasylkiw et al., 2012; Zessin, Dickhäuser, & Garbade, 2015; Zhu et al., 2019). In cancer specific populations, self-compassion has predicted reduced symptoms of depression and stress, and greater quality of life (Pinto-Gouveia, Duarte, Matos, & Fráguas, 2014). Self-compassion interventions have demonstrated efficacy in enhancing state self-compassion (Breines & Chen, 2012) and positive affect, and decreasing self-criticism and physiological arousal (Kirschner et al., 2019). Furthermore, meta-analyses have demonstrated self-compassion based interventions are effective in increasing trait self-compassion, and reducing body image (Turk & Waller, 2020) and psychological (Ferrari et al., 2019) distress.

‘My Changed Body’ (MyCB) is a self-compassion based writing activity developed to reduce BID in BCSs, by enhancing self-compassion (Przezdziecki, Alcorso, & Sherman, 2016). It involves a single expressive writing activity (EW; Pennebaker, 2000; Pennebaker, Kiecolt-Glaser, & Glaser, 1988), whereby participants are guided to write about a negative body image experience following breast cancer, followed by writing prompts with a self-compassion focus. Theoretically, self-compassion improves one’s body image, as applying a self-compassionate approach towards one’s body means perceiving one’s physical inadequacies in a balanced and kind way, rather than becoming overwhelmed or self-critical (Neff, 2003). Supporting this, a randomised study (Przezdziecki & Sherman, 2016) targeting BCSs (*N* = 152) found a paper-based version of MyCB significantly increased state self-compassion and was protective against increases in negative affect compared to active controls undertaking expressive writing (EW) alone (Pennebaker, 2000; Pennebaker et al., 1988). A randomised controlled trial (RCT) targeting BCSs (*N* = 306) demonstrated a web-based version of MyCB significantly reduced BID, and increased trait self-compassion and body appreciation, compared to active controls (EW). Women with lymphoedema in the MyCB condition also experienced significantly less depression and anxiety at follow-up compared to active controls (Sherman et al., 2018). However, the improvements in trait self-compassion and body image outcomes were most evident at 1-week follow-up, suggesting that to obtain a more robust improvement in self-compassion and body image outcomes a more intensive intervention approach may be required, such as the addition of other self-compassion directed components to the MyCB writing activity.

One promising addition could be self-compassion focused meditation, given it has been shown to effectively cultivate self-compassion regarding body image concerns (de Wet, Lane, & Mulgrew, 2020; Smeets, Neff, Alberts, & Peters, 2014). In a 3-week intervention involving daily self-compassion focused meditation, undergraduate participants (*N* = 220) reported reduced body dissatisfaction and body shame, with increased body appreciation and self-compassion (Albertson, Neff, & Dill-Shackleford, 2015). Meditation and writing are also combined in the multi-faceted Mindfulness based Self Compassion course (MSC), which has shown to increase self-compassion and reduce depression, anxiety and stress in non-cancer populations (Neff & Germer, 2013). To date, only one study has examined self-compassion-based meditation in an oncology population (Brooker et al., 2019). Promisingly, the MSC course increased participants’ body image satisfaction and reduced their avoidance, in a sample of cancer patients which included BCSs (Brooker et al., 2019).

Most of the research with BCSs has investigated the efficacy of related interventions, such as general compassion-based (e.g. Cognitively-Based Compassion Training) or mindfulness-based (e.g. Mindfulness-Based Stress Reduction) for improving mood and psychopathology. Such research suggests mindfulness-based (a core component of self-compassion) and compassion-based interventions, which include meditation as a key component, improve depression, stress, anxiety, mood and positive affect in BCSs, compared to controls (Bower et al., 2015; Boyle, Stanton, Ganz, Crespi, & Bower, 2017; Carlson et al., 2013; Dodds et al., 2015; Gonzalez-Hernandez et al., 2018; Hoffman et al., 2012). However, given the multi-faceted nature of these interventions, it is impossible to discern whether the meditation component was responsible for BCSs’ improvements in psychological distress. Further, all but one of these studies (Carlson et al., 2013), used wait-list, rather than active controls and thus cannot rule out alternative explanations, such as improved outcomes due to time in the intervention or expectations. Nevertheless, these studies suggest a self-compassion-based meditation intervention may improve psychological distress in BCSs. Notably, these interventions were delivered over a long period (typically involving 2 hour group sessions for 8 weeks, with additional daily home activities) in-person and are thus, time-intensive and costly. Hence, there are potential accessibility barriers for cancer survivors seeking psychological support (Grassi, Spiegel, & Riba, 2017; Tsaras et al., 2018). Online interventions overcome several barriers to engaging in face-to-face interventions, particularly geographical limitations and stigma (Mechael, 2009; Murray, 2012). Hence, accessible internet delivered self-compassion interventions such as MyCB, present a feasible alternative for the provision of support to BCSs. Indeed, there is increasing recognition of the need for research to develop less intensive, online-based interventions and compare these with more vigorous comparison groups, such as active controls (Haydon, Boyle, & Bower, 2018).

The overall aim of this pilot study was to assess the feasibility and efficacy of the online MyCB intervention for BCSs with the addition of a self-compassion meditation component compared with MyCB writing alone. A pilot study was undertaken, as while other research has investigated self-compassionate writing and meditation separately, or as part of a larger multi-faceted intervention, no study has examined these components together. It was predicted that compared to active (expressive writing) controls, individuals exposed to the MyCB writing with meditation or MyCB writing alone would report significantly lower BID at 1-month follow-up, and that the effect would be strongest for individuals receiving the additional meditation component. We further explored the impact of the MyCB conditions (with and without meditation) compared with controls on secondary outcomes including: self-compassion (trait and state), affect (positive and negative), body appreciation, and psychological distress (depression, anxiety and stress).

Given that few studies have thoroughly investigated the medical, demographic and psychological characteristics of BCSs that adhere to psychological interventions (Beatty et al., 2017; Beatty & Binnion, 2016), we further aimed to determine the uptake of the study and adherence to intervention activities, as well as to delineate characteristics of participants who were adherent to study protocols.

## Methods

### Participants

**Figure 1.**
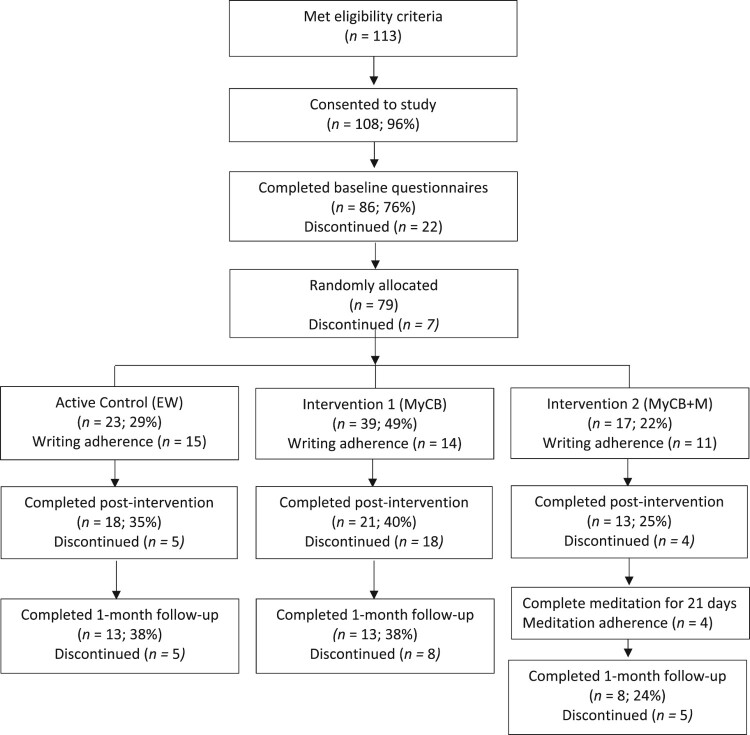
Participant flow diagram and recruitment numbers.

All recruitment took place during the time of COVID-19 (March to June 2020) and during the initial (first wave) restrictive lockdown that occurred across Australia, NZ and the UK. Participants were initially recruited from an Australian breast cancer consumer organisation, Breast Cancer Network Australia, and then extended to breast cancer consumer organisations in New Zealand (Shocking Pink), and the UK (Breast Cancer Now) to maximise recruitment opportunities during the COVID-19 pandemic. BCSs were eligible if they self-reported being: female; at least 18 years of age; had been diagnosed with stage I to III breast cancer, ductal carcinoma in situ (DCIS) and/or lobular carcinoma in situ (LCIS); had undergone breast cancer-related surgery; having experienced at least one negative event related to the changes that have occurred to their body after breast cancer (i.e. an event that has made them feel embarrassed, sad, angry, etc); and, could complete an online survey in English. Flow of participants through the study is provided in the CONSORT diagram (Figure 1).

Since this is a feasibility study, a formal sample size calculation is not required (Eldridge et al., 2016). However, to detect a small effect size with 0.90 power and an .05 critical alpha, it is recommended that pilot trials have at least 75 participants (25 per treatment arm) (Eldridge et al., 2016). Assuming an attrition rate of 15% at each timepoint, due to the study running during the COVID-19 pandemic, an initial sample of 100 participants is required at baseline for adequate power.

### Procedures

Between March and July, 2020 BCSs were invited to participate in the online study via Facebook advertisements and emails sent by each of the consumer organisations, which contained a link to the study website and consent form. Prior to accessing the consent form, participants were required to endorse being diagnosed with breast cancer and receipt of breast cancer related surgery. At 1-month follow-up, an email containing a link to access the questionnaire was sent to each participant. An additional two emails and one SMS reminder were sent if the questionnaire remained incomplete, unless participants requested to drop out of the study. After approximately 100 participants were recruited into the study, recruitment was ceased.

A three-arm parallel randomised controlled design with an active control, namely expressive writing with usual care (EW), and two versions of the self-compassion focused writing intervention, MyCB with usual care (MyCB) and MyCB plus meditation with usual care (MyCB + M) was used. After giving online consent, participants completed an online baseline questionnaire, containing measures of sociodemographic, medical and all psychological variables. They were then randomly assigned using the Qualtrics randomiser function to one of the three conditions in a ratio of 1:1:1. The research team and participants were blind to condition allocation. Additional questionnaires were completed immediately post-intervention (assessing state self-compassion and affect levels) and 1-month follow-up, containing measures of body image, psychopathology and trait self-compassion. The trial adhered to CONSORT requirements and ethics approval was granted by the Macquarie University Human Research Ethics Committee (approval Innumber 52020580814540). Informed written consent was obtained from each participant. The trial was registered with the Australian and New Zealand Clinical Trials Registry (#ACTRN12619001693112).

### Intervention and control conditions

Intervention group MyCB: Participants allocated to this condition completed a self-paced 6-step evidenced-based writing intervention that is estimated to take approximately 30 min (Sherman et al., 2018). Step 1 instructed participants to write freely about a negative body image experience related to their breast cancer. For steps 2–6 participants were prompted to: identify how they treated their body with kindness; provide kind advice to themselves; connect with others who may have had similar experiences; create awareness of their experience and response in a broader context; and, write a self-compassionate letter to themselves addressing the most salient points of their experience. Hence, the writing activity addresses mindful awareness, common humanity and self-kindness, as outlined by self-compassion research (Neff, 2016a).

Intervention group MyCB + M: This group completed the same writing activities as the MyCB group, in addition to listening to a brief 5-minute self-compassion based audio meditation (see supplementary file). Following completion of the initial MyCB writing, participants allocated to this condition were provided a link to download an audio file of a self-compassion based meditation, developed by the researchers and based on prior self-compassion research (Neff, 2019). The meditation audio-file was also emailed to participants so that it could be saved to their mobile or digital device for easy access. Participants were instructed to listen to the meditation either sitting or lying down, in a quiet and uninterrupted space, each day for the following three weeks, similar to Albertson et al. (2015). During this 3-week period, participants received a daily SMS reminder to maximise adherence to the meditation component.

Active Control EW: This condition entailed participants undergoing a writing task of similar length to the MyCB writing. The control group were requested to undertake expressive writing where they were prompted to write about a challenging body image experience as per the first step of the MyCB writing intervention (Pennebaker & Beall, 1986). Consistent with the MyCB writing, six writing prompts were provided for EW. However, the remaining prompts (Steps 2–6) excluded any self-compassion focused instructions and simply asked participants to continue exploring their experience, including event details, thoughts and feelings.

### Measures

#### Primary outcome

*Body Image Distress*. Body image distress was measured using the 10-item Body Image Scale (BIS: Hopwood, Fletcher, Lee, & Al Ghazal, 2001), validated in oncology populations. Participants indicate their agreement to statements (e.g. ‘Have you been dissatisfied with your appearance when dressed?’) on a 4-point Likert-type scale ranging from 0 *(not at all)* to 3 *(very much)* tapping into the cognitive, affective and behavioural aspects of body image disturbance. Total BID scores range from 0 to 30, with higher scores indicating greater BID. Importantly, the BIS has demonstrated adequate test re-test reliability (*r* = .70, *p* = .001) and sensitivity to change (*z* = −5.08, *p* < .001) over a 1 – and 3-month period, respectively (Hopwood et al., 2001). Further, the BIS has excellent internal reliability (Cronbach’s α = .93; Hopwood et al., 2001), including in the present study (Cronbach’s α = .94).

#### Secondary outcomes

*Body Appreciation*. The Body Appreciation Scale (BAS: Avalos, Tylka, & Wood-Barcalow, 2005) is a valid and reliable 13-item measure designed to assess positive aspects of body image. Participants indicate their agreement to statements (e.g. ‘I take a positive attitude towards my body’) on a 5-point Likert-type scale ranging from 1 *(never)* to 5 *(always).* BAS mean scores range from 1 to 5, with higher scores indicating greater body appreciation. Notably, the BAS has excellent test re-test reliability over a 3 week period (*r* = .90, *p* < .001; see Kling et al., 2019, for a review) and internal consistency (Cronbach’s α = .94; Avalos et al., 2005). While the BAS has not been validated in an oncology population, it has demonstrated excellent internal consistency in BCS samples (Cronbach’s α = .92-.94; Przezdziecki & Sherman, 2016; Sherman et al., 2018), including the current study (Cronbach’s α = .94).

*Self-Compassion*. State self-compassion was assessed using the 6-item self-compassionate attitude (SCA) measure developed by Przezdziecki and Sherman (2016). The scale reflects the definition of self-compassion (Neff, 2003) with the inclusion of body (Berry et al., 2010): mindful awareness of self (e.g. ‘connected with my emotions’), body self-acceptance, kindness and common humanity. Participants rate their current agreement with each statement on a 7-point Likert-type scale ranging from 1 (*not at all)* to 7 (*extremely*). Total SCA scores range from 7 to 42, with higher scores indicating greater state self-compassion. While the SCA scale has not been validated, it was developed for a BCS population and has demonstrated good internal consistency in BCS samples (Cronbach’s *α* = .85; Przezdziecki & Sherman, 2016) including the current sample (Cronbach’s *α* = .90).

Trait self-compassion was assessed using the valid and reliable 12-item Self-Compassion Scale – Short Form (SCS-SF: Raes, Pommier, Neff, & Van Gucht, 2011). The instrument consists of six subscales: mindfulness (e.g. ‘When something painful happens I try to take a balanced view of the situation.’), self-kindness, common humanity, isolation, self-judgement and over-identification. Participants indicate their agreement to each statement on a 5-point Likert-type scale ranging from 1 *(almost never)* to 5 *(almost always).* Items in the isolation, self-judgement and over-identification subscales are reverse coded. Total self-compassion scores range from 12 to 50 (Neff, 2016b; Neff, Whittaker, & Karl, 2017), with higher scores indicating greater self-compassion. The SCS-SF has a near perfect correlation with the full-scale (*r *= .95, *p* < .05; Raes et al., 2011), which has demonstrated excellent test re-test reliability (*r *= .93, *p* < .05; Neff, 2003) over a 3 week period. While the SCS-SF was validated on an undergraduate population, it has demonstrated good internal consistency in BCS samples (Cronbach’s *α* = .88; Sherman et al., 2018), including the present study (Cronbach’s *α* = .86).

*Positive and Negative Affect*. Affect was measured using the validated and reliable 20-item Positive and Negative Affect Schedule (PANAS: Watson, Clark, & Tellegen, 1988). The instrument comprises of two mood scales, one measuring positive affect (e.g. ‘Interested’, ‘Strong’) and negative affect (e.g. ‘Scared’, ‘Jittery’). Each item is rated on a 5-point Likert scale ranging from 1 *(very slightly or not at all)* to 5 *(extremely)* to indicate the extent to which the participant felt each item in the present moment. Total scores for each subscale range from 10 to 50, with higher scores representing greater levels of the respective affect. The PANAS was originally validated on an undergraduate sample and demonstrates adequate test re-test reliability over a 2-month period (Watson et al., 1988). It also demonstrates excellent internal consistency among BCS samples (Cronbach’s *α* = .88-92; Hall, Mishel, & Germino, 2014; Raque-Bogdan, Lent, & Lamphere, 2019), including the current sample (*α* = 0.92 for negative affect; *α* = 0.91 for positive affect).

*Depression, Anxiety and Stress*. The 21-item short form of the Depression, Anxiety and Stress Scale (DASS21) is a validated and reliable measure (Antony, Bieling, Cox, Enns, & Swinson, 1998; Cox, Antony, Enns, Swinson, & Bieling, 2005; Lovibond & Lovibond, 1995) consisting of three subscales assessing depression (e.g. ‘I found it difficult to work up the initiative to do things’), anxiety (e.g. ‘I felt I was close to panic’) and stress (e.g. ‘I find it hard to wind down.’). Participants rate each statement on a 4-point Likert-type scale ranging from 0 *(did not apply to me at all)* to 3 *(applied to me very much or most of the time).* Items on each subscale are summed, with subscale scores ranging from 0–21 and higher scores representing greater psychological distress. Validated on Australian samples (Antony et al., 1998; Lovibond & Lovibond, 1995; Ng et al., 2007), the DASS21 demonstrates sensitivity to change in clinical populations (*t* = 14.61-20.91; Ng et al., 2007). It also demonstrates good internal validity in BCS samples (Cronbach’s *α* = .78-.92; Przezdziecki et al., 2013; Sherman, Woon, French, & Elder, 2017), including the present sample(*α* = 0.91 for Depression; *α* = 0.83 for Anxiety; *α* = 0.88 for Stress).

#### Feasibility

Feasibility of the study was assessed by the uptake into the study, adherence to the study protocols and a user acceptability evaluation of the intervention activities.

*Uptake*. Measured as the number of participants who consented to the study and completed the baseline questionnaire as a proportion of those eligible.

*Adherence*. Adherence to the writing protocol was measured by the number of steps completed. This was defined as EW participants having completed at least the first prompt and MyCB participants completing all six prompts. Due to confidentiality, the content of the writing was not reviewed. Time taken to complete the writing activity online was not tracked. Participants in the MyCB + M group retrospectively self-reported the number of days to which the meditation was listened at 1-month follow-up, in response to the statement ‘How many days over the three weeks (21 days) did you listen to the meditation?’. Meditation adherence was defined as listening to the meditation at least 15 of the 21 days (i.e. 75%). Self-reported meditation adherence was not verified using objective measures.

*Evaluation of MyCB and Meditation*. A user acceptability measure was administered at the 1-month follow-up to measure the extent to which MyCB participants found the writing intervention and meditation (MyCB + M only) acceptable. Participants rated their agreement to each statement on a 6-point Likert-type scale ranging from 1 *(strongly disagree)* to 6 *(strongly agree).* Both MyCB groups rated their agreement to four statements regarding the writing activity (e.g. ‘The writing was appealing to me’). The MyCB + M group were asked to rate their agreement to an additional four statements of a similar nature (e.g. ‘The meditation was appealing to me’).

#### Demographic information

Demographic and medical information was collected including age, marital status, employment status, education level, breast cancer diagnosis, time since diagnosis, surgery type and complications.

### Data analysis

Descriptive statistics were used to identify demographic and medical characteristics of participants, by group and assess feasibility. Baseline differences between groups and adherers and non-adherers were analysed to determine possible covariates for later multivariate analyses using c^2^ tests of independence for categorical variables and one-way analysis of variance (ANOVA) for continuous variables. Fisher's exact test was used if any chi-square tests of independence cell count was less than 5. For continuous variables not meeting the assumption of normality, as assessed by Shapiro–Wilk’s (*p < *.05), or homogeneity of variances, as assessed by Levene’s Test of Homogeneity of Variance (*p* < .05), bootstrapping analyses were undertaken (1000 samples). Little’s missing at random (MCAR) was conducted for all outcome variables to confirm that any missing data were missing at random (χ^2^ = 46.02, df = 46, *p* = .47).

To assess the preliminary efficacy of the intervention two separate analyses were undertaken: (1) an intent-to-treat (ITT) analysis of all participants allocated to a condition; and, (2) a sensitivity analysis only including participants who adhered to the instructions for their allocated condition. In both instances, maximum-likelihood linear mixed models were used to test group, time and group by time interaction effects for all dependent variables, controlling for identified covariates. At post-intervention (i.e. after the writing activities and prior to exposure to the SC meditation for the MyCB + M group) analyses looked at the effects of the two MyCB interventions (MyCB and MyCB + M) compared with controls. For the 1-month analyses, comparisons were made across all three conditions. All analyses were carried out using SPSS version 23. An overall critical alpha of .05 was used for all analyses.

## Results

### Demographic, medical and psychological characteristics

Descriptive statistics of participant characteristics at baseline by condition, including relevant univariate and multivariate analyses comparing across groups are shown in Table 1. At baseline, most participants (52%) were diagnosed with stage I to III breast cancer, on average 7.28 years ago, and were on average 58.6 years old. In consideration of identifying potential covariates for later multivariate analyses, at baseline there were no significant differences on any demographic characteristics across conditions, with the exception of the number of breast surgery complications *F*(2,76) = 5.11, *p* = .01, and lymphoedema status *c^2^*(2, *N* = 79) = 6.50, *p* = .04. Follow-up analysis revealed that EW group participants reported significantly higher breast surgery complications than MyCB (*p* = .002) and MyCB + M (*p* = .036), and MyCB participants were less likely to have had lymphoedema than participants in EW (*p* = .008) and MyCB + M (*p* = .008).

**Table 1.** Participant characteristics at baseline by condition, presented as Mean (SD) or number of participants (%).

Characteristics	MyCB (*n =* 39)	MyCB + M (*n =* 17)	EW (*n =* 23)	*p*
	No. BCS (%)	No. BCS (%)	No. BCS (%)	
Age, years – mean (SD)	57.54 (13.64)	60.88 (8.42)	57.39 (9.53)	0.57
*Marital Status*				
Partnered	29 (47)	15 (24)	18 (29)	.59^+^
Not partnered	10 (56)	2 (12)	5 (29)	
*Recruitment Source*				
BCNA	28	9	20	.33^+^
Facebook	3	1	2	
Breast Cancer Now	2	3	0	
Other	6	4	1	
*Employment Status*				
Employed	21 (53)	6 (15)	13 (32)	0.35
Unemployed	18 (46)	11 (28)	10 (26)	
*Education*				
Less than high school^1^	11 (73)	2 (13)	2 (13)	.33^+^
Finished high school^1^	7 (39)	4 (22)	7 (39)	
Tertiary Qualifications	20 (44)	11 (24)	14 (31)	
*Stage*				
1	35 (47)	16 (22)	23 (31)	.18^+^
2 or 3	4 (100)	0 (0)	0 (0)	
*Cancer Diagnosis*				
Breast Cancer	21 (51)	7 (17)	13 (32)	0.57
DCIS/LCIS	17 (46)	10 (27)	10 (27)	
*Time since diagnosis, years*				
Mean *(SD)*	7.77 (6.73)	5.88 (3.78)	8.21 (5.43)	0.43
*Surgery Type*				
Mastectomy	23 (59)	13 (76)	19 (83)	.12^+^
Lumpectomy	16 (41)	4 (25)	4 (17)	
*Breast Surgery Complications*				
Mean *(SD)*	2.28 (1.95)	2.65 (2.64)	4.35 (3.16)	.01*
*Lymphoedema*				
Yes	4 (10)	2 (12)	8 (35)	.05*^+^
No	35 (90)	15 (88)	15 (65)	
*Hormone Therapy*				
Yes	17 (44)	8 (47)	8 (35)	0.7
No	22 (56)	9 (53)	15 (65)	
*Psychotherapy*				
Yes	3 (8)	4 (24)	5 (22)	.16^+^
No	36 (92)	13 (76)	18 (78)	
*Journal Writing*				
Yes	2 (5)	3 (18)	4 (17)	.22^+^
No	37 (95)	14 (82)	19 (83)	

* *p*<.05 ^+^ Fisher’s Exact utilised ^1^ High school or equivalent.

Regarding psychological outcome variables all outcome variables did not meet the assumption of normality or homogeneity variance, so bootstrapping (1000 samples) was applied to these ANOVAs. There were no significant differences in psychological variables at baseline between conditions (*p* range = .08 to .83), with the exception of body appreciation, *F*(2,76) = 4.95, *p* = .01. Follow-up analysis indicated that MyCB + M participants reported significantly higher body appreciation than EW (*p* = .03) and MyCB (*p* = .002). Subsequently, surgery complications, lymphoedema status and body appreciation were treated as covariates in all efficacy analyses.

### Efficacy of treatment

#### Intent to treat analysis

**Figure 2.**
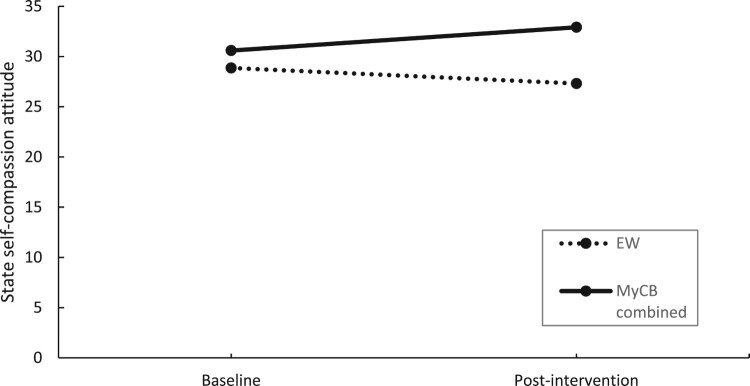
Changes in self-compassionate attitude over time by condition using ITT analysis.

Table 2 shows the time, condition, and time by condition effects at post-intervention using ITT analysis. There was a significant time by condition effect post-intervention, such that SCA scores in the MyCB groups (combined) significantly increased from baseline to post-intervention compared to EW, *F*(1,54) = 9.11, *p* < .01, *d* = 0.27 (Figure 2).

**Table 2.** Efficacy of treatment at post intervention using ITT analysis.

	MyCB combined (*n =* 56)	EW (*n =* 23)	Main Effect Time	Main Effect Group	Group × Time Interaction	
	*M (SD)*	*M (SD)*	*p/95% CI*	*p/95% CI*	*F*	*p/95% CI*
*Self-Compassionate attitude*						
Baseline	30.60 (9.10)	28.87 (7.35)	.55 (−3.84, 0.82)	.04 (−9.48, −1.74)	9.11	<.01* (1.30, 6.46)
Post Intervention	32.92 (9.69)	27.32 (7.67)				
*Positive Affect*						
Baseline	32.45 (12.19)	31.05 (9.94)	.88 (−3.49, 1.28)	.33 (−8.62, 2.04)	.86	.36 (−2.20, 5.99)
Post Intervention	33.55 (13.28)	30.26 (10.53)				
*Negative Affect*						
Baseline	16.48 (8.63)	16.83 (7.04)	.49 (−0.74, 2.61)	.64 (−2.53, 5.00)	.38	.54 (−3.75, 1.98)
Post Intervention	15.55 (9.39)	16.78 (7.44)				

Note*:* Estimated marginal means and standard deviations reported. Controlling for age, Lymphoedema status and total complications reported at baseline.* *p*<.05.

Table 3 shows the time, condition, and time by condition effects at 1-month follow-up using ITT analysis. A significant main effect of time was obtained for the primary outcome of BID, in which scores were significantly lower across all conditions at 1-month follow-up compared to baseline, *F*(1,36) = 5.79, *p* = .02. A significant main effect of time was also obtained for trait self-compassion, such that scores were significantly higher across all conditions at 1-month follow-up compared to baseline, *F*(1,38) = 4.35, *p* = .04.

**Table 3.** Efficacy of treatment at 1-month follow-up using ITT analysis.

	MyCB (*n =* 39)	MyCB + M (*n =* 17)	EW (*n =* 23)	Main effect time	Main effect group	Group × Time interaction	
	*M (SD)*	*M (SD)*	*M (SD)*	*p/95% CI*	*p/95% CI^a^*	*F*	*p/95% CI^a^*
*Body Image Distress*							
Baseline	12.07 (8.62)	10.65 (7.67)	13.57 (7.30)	.02* (0.61, 11.39)	.83 (−5.27, 4.74) (−5.24, 3.91)	2.25	.12 (0.14, 6.25) (−0.96, 5.13)
1-month follow-up	10.33 (9.87)	10.65 (7.67)	10.72 (8.00)				
*Body Image Appreciation*							
Baseline	3.33 (8.74)	3.79 (0.78)	3.18 (0.77)	.38 (−0.56, 0.48)	.05 (−0.96, 0.30) (−1.12, 0.11)	.72	.49 (−0.94, 0.39) (−0.61, 0.70)
1-month follow-up	3.32 (1.31)	3.82 (1.03)	3.49 (0.96)				
*Self-Compassion (trait)*							
Baseline	39.78 (10.80)	40.47 (9.55)	36.92 (9.22)	.04* (−7.67, −0.22)	.29 (−11.95, 1.08) (−10.97, 1.15)	1.73	.19 (−2.86, 6.62) (−0.49, 8.94)
1-month follow-up	39.50 (13.1)	44.42 (10.88)	38.98 (10.33)				
*Depression*							
Baseline	3.43 (4.80)	2.95 (4.25)	3.16 (0.41)	.96 (−1.35, 1.80)	.74 (−2.71, 3.05) (−1.53, 3.79)	.39	.68 (−1.96, 2.04) (−1.53, 3.79)
1-month follow-up	3.86 (5.74)	2.73 (4.82)	2.90 (4.56)				
*Anxiety*							
Baseline	2.49 (3.62)	3.97 (3.22)	2.58 (3.11)	.46 (−0.83, 2.24)	.38 (−3.16, 1.46) (−2.96, 1.41)	.27	.76 (−2.50, 1.43) (−2.65, 1.25)
1-month follow-up	2.49 (4.68)	3.26 (3.83)	2.41 (3.65)				
*Stress*							
Baseline	4.80 (5.06)	5.71 (4.04)	5.95 (3.88)	.94 (−1.96, 1.92)	.63 (−2.90, 2.91) (−3.39, 2.11)	.11	.90 (−2.24, 2.70) (−2.72, 2.18)
1-month follow-up	5.09 (5.93)	5.73 (4.74)	5.71 (4.56)				

Note: Estimated marginal means and standard deviations reported. Controlling for age, Lymphoedema status and total complications reported at baseline.

* *p*<.05.

#### Sensitivity analysis

**Figure 3.**
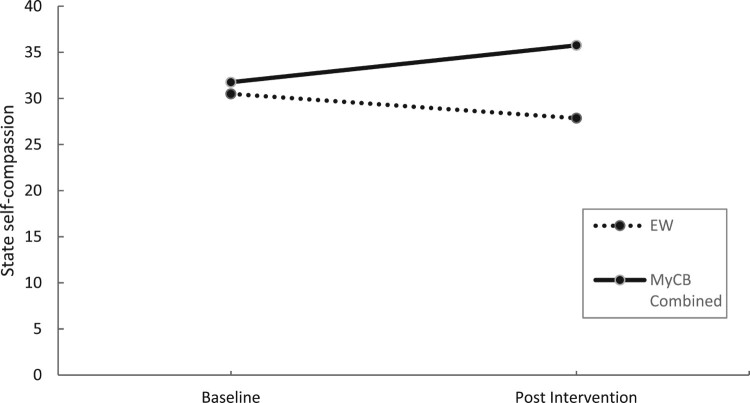
Changes in self-compassionate attitude over time by condition using sensitivity analysis.

**Figure 4.**
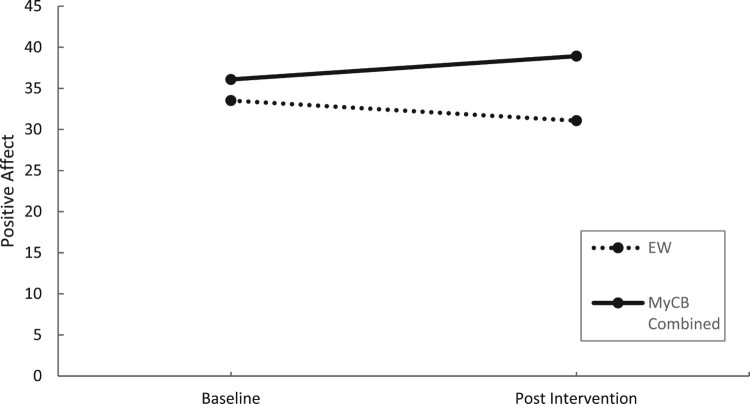
Changes in positive affect over time by condition using sensitivity analysis.

Sensitivity analysis (Table 4) showed a significant time by condition interaction effect at post intervention for self-compassionate attitude, *F*(1,23) = 12.10, *p* = .002, *d* = 0.95 (Figure 3) and positive affect, *F*(1,23) = 4.34, *p* = .046, *d =* 0.83 (Figure 4), with both variables significantly increasing from baseline to post-intervention for MyCB (Combined group) compared to EW.

**Table 4.** Efficacy of treatment at post intervention using sensitivity analysis.

	MyCB (combined) (*n =* 12)	EW (*n* = 11)	Main effect time	Main effect group	Group × Time interaction	
	*M (SD)*	*M (SD)*	*p*	*p*	*F*	*p*
*Self-Compassionate attitude*						
Baseline	31.76 (8.43)	30.50 (8.18)	.48 (−6.73, 1.27)	.15 (−14.48, 1.32)	12.10	<.01* (2.69, 10.58)
Post Intervention	35.76 (8.43)	27.86 (8.18)				
*Positive Affect*						
Baseline	36.08 (9.54)	33.52 (9.26)	.88 (−6.43, 0.76)	.14 (−15.30, 0.41)	4.43	<.05* (0.09, 10.48)
Post Intervention	38.92 (9.54)	31.07 (9.26)				
*Negative Affect*						
Baseline	14.98 (8.14)	16.68 (8.02)	.79 (−2.49, 5.66)	.32 (−2.35, 10.55)	.71	.41 (−8.29, 3.49)
Post Intervention	13.40 (8.14)	17.50 (8.02)				

Note: Means and standard deviations reported. Controlling for age, Lymphoedema status and total complications reported at baseline.

* *p*<.05.

**Figure 5.**
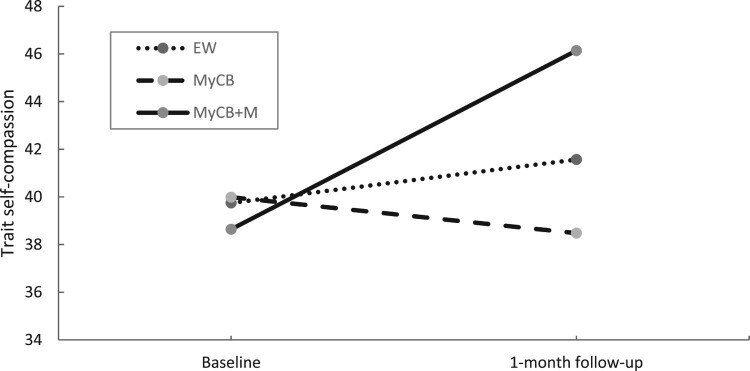
Changes in trait self-compassion over time by condition using sensitivity analysis.

**Figure 6.**
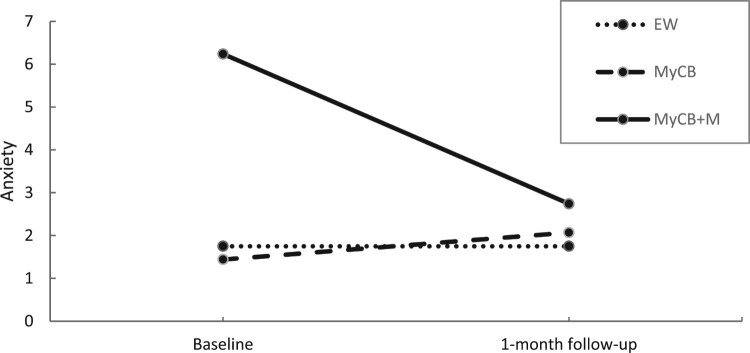
Changes in anxiety over time by condition using sensitivity analysis.

At 1-month follow-up, sensitivity analysis (Table 5) showed a significant main effect of time for the primary outcome of body image distress, *F*(1,23) = 8.19, *p* = .009, in which scores significantly reduced at 1-month follow-up compared to baseline. A significant time by condition effect was found for trait self-compassion, *F*(2,23) = 3.65, *p* = .042 (Figure 5). Follow-up analysis showed trait self-compassion scores significantly increased for the MyCB + M group over time compared to MyCB *t*(23) = 2.70, *p* = .013, *d* = 0.74. No significant differences between groups were found. A significant time by condition effect was also found for anxiety, *F*(2,23) = 8.12, *p* = .002 (Figure 6). Follow-up analysis showed anxiety scores significantly reduced over time for the MyCB + M group compared to EW, *t*(23) = −3.464, *p* = .002, *d* = 0.31, and MyCB, *t*(23) = −3.893, *p* = .001, *d* = 0.22. There were no significant differences between EW and MyCB.

**Table 5.** Efficacy of treatment at 1-month follow-up using sensitivity analysis.

	MyCB (*n =* 8)	MyCB + M (*n =* 4)	EW (*n =* 11)	Main Effect Time	Main Effect Group	Group x Time Interaction	
	*M (SD)*	*M (SD)*	*M*	*p/95% CI*	*p/95% CI^a^*	*F*	*p/95% CI^a^*
*Body Image Distress*							
Baseline	10.99 (7.44)	11.42 (6.19)	14.56 (7.35)	<.01* (1.97, 3.97)	.74 (−7.30, 8.44) (−8.26, 6.91)	1.69	.21 (−1.11, 5.84) (−3.39, 3.89)
1-month F/U	9.75 (7.44)	10.42 (6.19)	10.99 (7.35)				
*Body Appreciation*							
Baseline	3.48 (.64)	3.98 (.53)	3.37 (.63)	.13 (−0.42, 0.26)	.20 (−1.21, 0.151) (−1.11, 0.21)	.08	.92 (−0.48, 0.32) (−0.47, 0.37)
1-month F/U	3.61 (.64)	4.06 (.53)	3.53 (.63)				
Trait Self-Compassion							
Baseline	39.99^1^ (10.89)	38.64^2^ (9.06)	39.75^1,2^ (3.25)	<.05* (−13.13, −1.87)	.83 (−16.13, 6.99) (−18.81, 3.52)	3.65	.04* (0.90, 12.26) (2.10, 15.90)
1-month F/U	38.48 (10.89)	46.14 (9.06)	41.57 (3.25)				
*Depression*							
Baseline	2.26 (4.13)	3.99 (3.42)	2.80 (4.09)	.76 (−0.99, 2.99)	.88 (−4.48, 4.27) (−4.46, 3.99)	.82	.45 (−3.41, 1.23) (−3.93, 0.93)
1-month F/U	2.76 (4.13)	2.99 (3.42)	2.89 (4.09)				
*Anxiety*							
Baseline	1.44 ^1^ (3.20)	6.24^2^ (2.66)	1.75^1^ (3.32)	.02* (1.71, 5.29)	.17 (−4.37, 2.40) (−6.32, 1.93)	8.12	<.01* (−5.59, −1.41) (−6.32, −1.93)
1-month F/U	2.07 (3.20)	2.74 (2.66)	1.75 (3.32)				
*Stress*							
Baseline	5.16 (4.44)	7.04 (3.73)	5.67 (4.39)	.36 (−0.87, 5.37)	.92 (−3.64, 5.78) (−4.08, 5.07)	1.05	.37 (−6.08, 1.21) (−6.20, 1.45)
1-month F/U	5.28 (4.44)	4.77 (3.73)	5.86 (4.39)				

Note: Adjusted means and standard deviations reported. Controlling for age, lymphoedema status and total complications reported at baseline. ^a^Confidence intervals are reported for contrasts: EW vs. MyCB + M, MyCB vs. MyCB + M

^1,2^ Denotes contrasts conducted following significant group x time interaction: same number indicates no significant difference, and different numbers indicate significant difference. * *p*<.05.

### Study uptake and adherence

In total, 113 individuals meeting eligibility criteria visited the research study website. Of these, 108 people consented (86%), and 79 participants (70% uptake) completed baseline questionnaires. These participants were then randomly assigned to study conditions, MyCB (*n* = 39), MyCB + Meditation (MyCB + M) (*n* = 17) and EW (*n* = 23). Forty participants adhered to their respective writing protocols at baseline (51%), including 15 in the active control condition (EW) and 25 in the MyCB condition (MyCB and MyCB + M combined). At 1-month follow-up, 34 (31%) of consenting participants completed the study questionnaire and four participants reported adhering to the meditation protocol. Twenty-three (21%) of participants completed all questionnaires and adhered to all their respective protocols. Three participants personally emailed the researchers to explain their withdrawal from the study. One expressed a concern about filling out the questions during the pandemic as she felt her mental health had been dramatically impacted by national lockdowns. The other two found it difficult to write about a negative body-related event, reporting it brought up a lot of uncomfortable emotions.

### Factors associated with adherence

Characteristics of participants that adhered to writing protocols using univariate and multivariate analysis are shown in Table 6. Factors significantly associated with writing adherence across all conditions, were: older age (*p* = .003); higher baseline scores in body appreciation (*p* = .026), trait self-compassion (*p* = .028) and state self-compassionate attitude (*p* = .0.25); and lower scores in negative affect (*p* = .005) and depression (*p* = .021). Factors significantly associated with writing adherence in the MyCB condition were older age (*p* = .006); not having lumpectomy surgery (*p* = .046); less breast surgery complications (*p* = .043); higher scores in body appreciation (*p* = .009) and trait self-compassion (*p* = .045); and lower scores in body image distress (*p* = .027), negative affect (*p* = .005) and depression (*p* = .021). There were no significant differences between MyCB + M adherers and non-adherers (values ranged from *p = *.22 to .89).

**Table 6.** Characteristics of participants that completed writing at baseline and adhered to MyCB Protocol, presented as Mean (SD) or number (%) of participants.

Characteristics	Completed writing (all groups) (*n =* 79)			MyCB (combined) (*n* = 56)		
	Discontinued (*n* = 39)	Remained (*n* = 40)	*p*	Non Adherers (*n* = 31)	Adherers (*n* = 25)	*p*
*Demographic*						
Age, years	54.41 (12.26)	61.93 (9.51)	<.01*	54.58 (12.52)	63.48 (10.28)	<.01*
Resides in Australia^a^	26 (45.61)	31 (54.39)	0.42	20 (51.28)	19 (48.72)	.73^+^
Partnered^a^	32 (51.61)	30 (48.39)	0.45	24 (54.55)	20 (45.45)	0.82
Employed^a^	21 (52.50)	19 (47.50)	0.57	17 (62.96)	10 (37.04)	0.27
Tertiary Education^a^	22(49)	23(51)	0.88	18(58)	13(42)	0.77
*Cancer Diagnosis^a^*						
Breast Cancer	20 (48.78)	21 (51.22)	0.82	16 (57.14)	12 (42.86)	0.91
DCIS /LCIS	19 (51.35)	18 (48.65)		15 (55.56)	12 (44.44)	
Stage (1)	35 (47)	39 (53)	.65^+^	29 (56.86)	22 (43.14)	.59^+^
Time since diagnosis, years	6.35 (4.06)	8.61 (7.03)	.07^	5.89 (4.03)	8.82 (7.6)	.07^
*Surgery Type*						
Mastectomy^a^	22 (40)	33 (60)	0.15	16 (44.44)	20 (55.56)	0.47
Lumpectomy^a^	17 (70.81)	7 (29.17)	0.06	15 (75)	5 (25)	<.05*
Breast Surgery Complications	2.92 (2.41)	3 (2.85)	.90^	2.87 (2.48)	1.8 (1.53)	.04*^
Lymphoedema^a^	6 (42.80)	8 (57.14)	.59^+^	27 (54)	23 (46)	.44^+^
Hormone Therapy^a^	15 (45)	18 (54)	0.56	13 (52)	12 (48)	0.65
Psychotherapy^a^	6 (50)	6 (50)	.60^+^	27 (55)	22 (45)	.62^+^
Journal Writing^a^	1 (11)	8 (89)	.03^+^	1 (20)	4 (80)	.16^+^
*Psychological*						
Body Image Distress	12.36 (9.38)	10.05 (8.04)	.22^	11.94 (.10)	7.32 (6.69)	.03*^
Body Image App	3.26 (.89)	3.68 (.74)	.03*	3.33 (.94)	3.92 (.61)	<.01*
Trait Self-Compassion	36.92 (8.84)	41.40 (9.18)	.03*	29.97 (7.95)	42.48 (7.11)	<.05*
State Self-Compassion	28.95 (7.78)	32.63 (6.40)	.03*	38.07 (8.56)	33.68 (5.99)	0.06
Positive Affect	31.00 (9.75)	32.93 (7.76)	0.33	31.52 (10.32)	33.88 (7.1)	.34^
Negative Affect	17.12 (7.43)	14.60 (6.02)	&lt;01*^	17.58 (7.70)	13.4 (4.2)	<.01*^
Depression	4.21 (4.60)	2.45 (3.08)	.02*^	4.23 (4.68)	1.96 (2.52)	.02*^
Anxiety	2.82 (3.44)	2.28 (2.83)	.53^	2.68 (3.35)	2.16 (2.82)	.52^
Stress	6.36 (4.26)	4.35 (3.63)	.08^	5.74 (4.06)	3.93 (3.72)	.06^

^a^ Reflects the number and percentage of participants answering ‘yes’ to this category.

* *p* < 0.05.

^ Transformed data utilised.

^+^ Fisher’s Exact utilised.

### User acceptability evaluation of MyCB and meditation

Descriptive statistics of user acceptability evaluation of MyCB and the meditation are shown in Table 7. The majority of MyCB participants agreed (somewhat to strongly) that the writing activity appealed to them (79%), they were comfortable with the writing activity (74%), instructions were easy to understand (79%) and they would be happy to do the writing activity again (68%). The majority of MyCB + M participants strongly agreed the meditation appealed to them (75%) and would be happy to listen to the meditation again (63%). The majority (88%) also agreed (mostly to strongly) that the instructions were easy to understand, and 50% agreed they were comfortable listening to the meditation.

**Table 7.** Evaluation questions and responses.

Questions	MyCB* (*n* = 19)	Meditation (*n =* 8)
Number of participants that ‘somewhat’ to ‘strongly’ agreed with the following statements		
This writing activity/meditation was appealing to me	15 (79%)	6 (75)
I felt comfortable doing the writing activity / listening to the meditation	14 (74%)	4 (50)
I found the writing / meditation instructions easy to understand	15 (79%)	7 (88)
I would be happy to do the writing activity / meditation again	13 (67%)	5 (63)

* Includes MyCB and MyCB + M participants

## Discussion

The primary aim of this pilot study was to examine the efficacy of a brief web-based self-compassion-based writing intervention (MyCB) with the addition of a self-compassion meditation component for BSCs. A secondary aim of this study was to ascertain the feasibility and acceptability of this type of online study compared with prior studies of this intervention approach. In terms of preliminary evidence for efficacy of the MyCB approach with the additional component of meditation, the findings were mixed. Consistent with prior research (Przezdziecki & Sherman, 2016; Sherman, Roper, & Kilby, 2019) the MyCB writing activity led to enhanced self-compassionate outlook immediately post-writing compared with those undertaking expressive writing. Moreover, enhancements in positive affect for the MyCB groups immediately following the writing intervention are consistent with prior research in non-oncology populations demonstrating that writing in a self-compassionate way improves mood (Leary, Tate, Adams, Allen, & Hancock, 2007; Neff, Toth-Kiraly, Knox, Kuchar, & Davidson, 2021; Odou & Brinker, 2014). These findings provide clinical utility as they suggest encouraging individuals to cultivate a self-compassionate attitude after experiencing a negative event may promote better coping (Johnson & O’Brien, 2013) and improve mood, which can be rapidly achieved with a brief writing exercise (Adams & Leary, 2007).

In terms of the relative effect of the additional self-compassion meditation component to the MyCB writing activity, we observed an enhancement in a few of the study outcomes. Specifically, 1-month follow-up sensitivity analysis revealed that the meditation group demonstrated significant enhancements in trait self-compassion, with a large effect size, compared to self-compassionate writing alone (MyCB), suggesting additional and regular exposure to self-compassionate messaging may lead to a more sustained effect on self-compassion. Additionally, individuals in the meditation condition reported reduced anxiety at 1-month follow-up compared to both the MyCB and EW conditions, adding further support to the growing body of research advocating that self-compassion based activities, such as meditation, can cultivate self-compassion and reduce psychological distress (Ferrari et al., 2019). Notably, the meditation group demonstrated a larger improvement in anxiety at 1-month follow-up relative to the EW group (*d *= 0.31) than what was obtained in the prior large RCT (*d* = 0.14) of MyCB intervention (Sherman et al., 2018), further supporting the incremental value of the meditation component for treating psychological distress. Such findings have important clinical utility given the brevity of the interventions, ease of dissemination via the internet and that cancer patients are at an increased risk of experiencing anxiety (Tsaras et al., 2018), which can impede treatment and quality of life (Caruso, Nanni, Riba, Sabato, & Grassi, 2017). The superior outcomes for the meditation group may be explained by the additional practice effects (an additional 21 days of self-compassion meditation practice) providing cumulative benefits (Albertson et al., 2015; Neff & Germer, 2013). However, it is prudent to note that only four participants adhered to the meditation protocol and therefore other alternative explanations cannot be ruled out. Surprisingly, this study found using ITT analysis that by 1-month follow-up, participants in all writing conditions experienced improvement in trait self-compassion, contrary to prior MyCB research with BCSs, which found superior improvements in trait self-compassion for participants in the self-compassionate writing condition compared with EW (Sherman et al., 2018). This may be explained by the questionnaires, particularly the self-compassion measures, requiring a degree of awareness, self-reflection and connection with self, which are elements of being self-compassionate (Neff, 2016). It is possible that simply answering the questions prompted a shift in self-compassion.

Another surprising finding of this study was that by 1-month follow-up participants in all writing conditions experienced a reduction in BID, contrary to prior MyCB research with BCSs reporting superior improvements in body image after self-compassion writing compared with expressive writing controls (Sherman et al., 2018). This finding suggests that the act of disclosing in writing about one’s innermost thoughts regarding breast cancer-related body image concerns in itself has had a facilitatory effect on body image, whether guided self-compassionately or not. One study within the disordered eating context found similar facilitatory effects of writing disclosure on body image were likely due to the expressive writing operating by activating positive attitudes towards oneself (O’Connor et al., 2011), a mechanism similar to that which has been purported for enhancement of self-compassion. Yet, there is other preliminary evidence suggesting that the very act of writing of any kind may improve body image (Earnhardt, Martz, Ballard, & Curtin, 2002). An alternate explanation for the lack of significant differences in body image between the MyCB conditions and control could simply be a reflection of the sample size in the present study, which was considerably lower compared to those previously reported (Sherman et al., 2018) and hence, likely to be underpowered to detect an effect. Future research should seek to disentangle the possible effects evident here from the active control EW condition by including a ‘benign’ control condition, such as a waitlist. Furthermore, in the prior research BID was on average worse for participants entering the study than in the present study, and the greatest effects of MyCB writing were obtained with those participants experiencing lymphoedema who had the greatest BID at study entry (Sherman et al., 2018).

This is the first known study to investigate uptake and adherence of a web-based psychosocial intervention for BCSs during a global pandemic, adding to the limited research in the area. Overall, study uptake was 76% of eligible individuals, which is higher than the 50% uptake reported in a meta-analysis of psychological interventions targeting cancer survivors (Brebach, Sharpe, Costa, Rhodes, & Butow, 2016), yet lower than previously reported in MyCB-related research (Sherman et al., 2018). Adherence to each of the MyCB (45%) and EW (50%) writing activities was modest and lower than reported in prior MyCB research (MyCB: 88% vs. EW: 81%) (Sherman et al., 2018), yet comparable with completion rates of 40-70% of other online psychological interventions targeting cancer and non-cancer populations (Beatty et al., 2017; Donkin et al., 2011). Self-reported adherence to the meditations (50%) was similar to that of the other conditions in this study and to self-compassion meditation adherence previously documented Albertson et al. (2015).

Participants were more likely to adhere to either of the writing activities if they were older, had greater body appreciation and self-compassion, and less negative affect and depression at the time of study enrolment. Moreover, participants more likely to adhere to the self-compassion writing (MyCB) had additionally experienced fewer breast surgery complications, less BID and less likely to have had a lumpectomy at study entry. This is consistent with a prior study reporting that older age predicts adherence to online interventions targeting cancer related distress (Beatty et al., 2017), and further adds to the mixed findings regarding adherence and baseline symptom severity (Beatty & Binnion, 2016). However, these findings unfortunately suggest BCSs experiencing higher symptom severity and lower self-compassion at the point of entering the study were less likely to complete either writing activity. Since younger BCSs are more likely to experience psychological distress (Helms et al., 2008), this may help explain why adherers were both older and less distressed than non-adherers in this study. Moreover, uptake and adherence may have been impeded in this study by what is referred to as ‘fear of self-compassion’ characterised by feelings of not deserving compassion or perceiving it as weak (Gilbert, McEwan, Matos, & Rivis, 2011), which is prevalent in chronic mental health patients (Gilbert & Procter, 2006) and trauma populations (Boykin et al., 2018). This initial resistance to being self-compassionate has previously been addressed using psychoeducation (Gilbert & Procter, 2006), which may be a critical component to be incorporated into future applications of the MyCB intervention approach. Psychoeducation dispelling myths and fears around treating oneself self-compassionately may be particularly helpful for improving adherence among younger BCSs, who tend to report lower self-compassion (Przezdziecki et al., 2013; Przezdziecki & Sherman, 2016; Todorov, Sherman, & Kilby, 2019), perhaps due to a fear of being self-compassionate. Alternatively, an intervention which more directly addresses the importance that younger BCSs place on physical appearance for their self-worth (self-evaluative salience), may be more beneficial for younger BCSs, who typically struggle with this evaluative component of body image (Moreira & Canavarro, 2010; Sherman et al., 2017). In terms of acceptability, the majority of MyCB and Meditation participants agreed the interventions were appealing, easy to understand and they would be happy to undertake the activities again. Similarly, the majority of participants indicated they were comfortable undertaking the MyCB writing activity, yet for the Meditation component this was split with only half of the participants feeling comfortable with this approach. Overall, this suggests a high level of acceptability for self-compassion intervention approaches.

Taken together, these findings support the user acceptability of self-compassionate writing intervention approaches such as MyCB (Przezdziecki et al., 2016) and provide preliminary evidence for the efficacy of incorporating an additional self-compassion based meditation component to enhance the impacts of this intervention. This adds further support to the clinical use of self-compassion interventions within the breast cancer context, and in particular for the use of writing-based interventions to address body image related issues in BCSs. The study demonstrated several strengths, including being a registered RCT, the utilisation of an established self-compassion based writing activity (MyCB) and an active control. The use of the active control was a distinct strength of the study.

In consideration of these findings, several limitations need to be considered in the context of opportunities for future research in this area. The small sample size and hence lack of power are limitations, along with the unique historical context of intervention participation, occurring during the height of the COVID-19 pandemic, and thus limiting the generalisability of the findings to non-pandemic contexts. However, the study provides a unique insight into the potential role of the COVID-19 pandemic in intervention adherence. Emerging research suggests uptake of mental health support has reduced during the COVID-19 pandemic in the general population and in BCSs (Shinan-Altman, Levkovich, & Tavori, 2020; Yao, Chen, & Xu, 2020). However, little is known about the impact the pandemic has on recruitment into online intervention studies. Adherence may be related to lower baseline symptom severity, as demonstrated by the current study and one previous study of the general public in China (Yao et al., 2020). This suggests that in the current health crisis, to recruit individuals who are experiencing more symptom severity (i.e. body image distress) into the MyCB study, greater awareness and education is required regarding the potential benefits of self-compassion during the recruitment process. It is especially important that we look for ways to improve online intervention recruitment and adherence, given the current global pandemic and the restrictions placed on face-to-face interactions.

Another potential limitation in the present study was that state self-compassion was measured using a published but non-validated measure (Przezdziecki & Sherman, 2016). In light of the development of a new reliable and validated state measure of self-compassion (Neff et al., 2021) (State Self-Compassion Scale), future self-compassion research in the MyCB context should consider use of this measure. Moreover, despite the improvements in state self-compassion immediately post-intervention and trait self-compassion at 1-month follow-up, further research is required to ascertain the mechanisms by which self-compassion as an attitude becomes an enduring trait.

This study explored effects at 1-month follow-up, however the longer-term efficacy of the intervention is unknown. Previous research demonstrated efficacy of MyCB on reducing BID and increasing body appreciation in BCSs at 1-month follow-up, with body appreciation effects maintained at 3-months (Sherman et al., 2018). Furthermore, effects were moderated by lymphoedema and body appearance investment and improvements in BID and body appreciation at 1 and 3-month follow-up were mediated by self-compassion (Sherman et al., 2018). Future studies involving MyCB and MyCB + M should seek to replicate these findings. Furthermore, acceptability and efficacy (using sensitivity analysis) should be interpreted with caution as it is possible that only participants that perceived the programme as beneficial and acceptable remained at 1-month follow-up, raising the question of whether these findings are generalisable to all BCSs. Although, there is increasing support for psychological interventions being tailored to suit cancer survivors’ specific needs and preferences (Corbett et al., 2018).

While having experienced at least one negative body image-related event following breast cancer was listed as part of the study inclusion criteria and self-reported by participants, it was not explicitly screened for. Further, no minimum level of BID was required and was relatively low at baseline (*M* = 11.17 out of a possible 30). Despite data collection occurring during the height of the COVID-19 pandemic (March to July, 2020), a time of increased rates of psychopathology for many (Wang et al., 2020)*,* the sample were remarkably well adjusted. In comparison with pre-COVID-19 BCS samples, the present sample at baseline reported similar levels of positive affect, BID, body appreciation and state self-compassion, and surprisingly significantly lower negative affect with a large effect size (Przezdziecki & Sherman, 2016; Raque-Bogdan et al., 2019; Sherman et al., 2018; Todorov et al., 2019). Two recent studies have similarly reported that cancer patients are coping well with the pandemic (Frey et al., 2020; Louvardi et al., 2020), yet there may be somewhat of a self-selection bias with studies conducted during COVID-19, as those who volunteer for research during a global health crisis are likely well-adjusted. Sherman et al. (2018) found MyCB benefitted participants that were experiencing higher distress. Hence, future research should target these women.

Notably, in spite of ethics approval to directly recruit from breast clinics this was not possible during the COVID-19 outbreak (Shinan-Altman et al., 2020) and so recruitment was conducted entirely online, restricting the sample to Internet users, who were actively seeking treatment and members of online-community breast cancer groups. Being a part of these online community groups may have provided a source of social support (Classen et al., 2008; Giese-Davis et al., 2002) for participants during the pandemic, perhaps explaining why the sample was so well-adjusted at baseline. The self-selection process regarding recruitment into this study is both a strength and a weakness. Recruiting individuals through social media and online support groups may limit the characteristics of individuals likely to participate in such an online study. Yet a distinct strength of the recruitment approach was the inclusion of participants from multiple countries, a strategy adopted by the researchers to enhance study uptake during the COVID-19 pandemic. In spite of the breadth of recruitment, the majority of participants were Australian, partnered, had received tertiary education and due to the study design, are computer literate with internet access. Hence, future research should look to expand access to other BCS populations. Additionally, this study recruited participants who had been diagnosed with breast cancer across a large range of time since diagnosis (between 6 months and 31 years). Hence, more research is required to determine the optimal time following diagnosis and treatment to administer self-compassionate interventions to support BCSs experiencing distress. A meta-analysis found patient uptake into such online intervention studies is typically greater when offered closer to the point of diagnosis and by a nurse at the clinic (Brebach et al., 2016), suggesting future research should consider these alternate recruitment strategies.

Finally, in respect of participant confidentiality, the content of writing was not reviewed, and thus it was not possible to ascertain adherence to the MyCB writing prompts. Further, meditation adherence was via self-report. It would be prudent to include a manipulation check in future research whereby participants detail what was outlined in the meditation. Time taken to complete the writing and participants’ word count was not recorded, nor was the quantity of meditation practice, precluding analysis of these more objective measures of intervention adherence with study outcomes. Lastly, meditation acceptability was solely assessed through quantitative measures. Future research should also examine user acceptability with qualitative methods to gain a more in-depth understanding of participants’ intervention experiences. In conclusion, this study demonstrates the preliminary efficacy of a brief self-compassion based writing and meditation activity, and lends greater support to MyCB being integrated into BCSs stepped-care initiatives to increase self-compassion and psychological wellbeing. It also highlights the importance of increasing promotion and psycho-education of self-compassion and mental health initiatives to support those experiencing heightened distress, particularly during a global crisis when mental health intervention uptake is reduced. While the small sample size and self-report nature of this study preclude a more vigorous examination of the meditation component’s feasibility and efficacy, the findings suggest the meditation may be a promising adjunct to MyCB. With an additional psychoeducation component, the intervention may be able to recruit more distressed BCSs and thus, have an even bigger impact on BCS psychological adjustment.

## Supplementary Material

Supplemental Material

Supplemental Material
