# Supplementary material for: Feasibility and pilot study of a brief self-compassion intervention addressing body image distress in breast cancer survivors
Source: Health Psychol Behav Med. 2021 May 21;9(1):498–526. doi: 10.1080/21642850.2021.1929236 (PMC8158280; doi:10.1080/21642850.2021.1929236)
Supplement: Supplemental Material [file RHPB_A_1929236_SM0816.docx]

**Self-Compassion Meditation Script**

Find yourself in a comfortable position laying down or seated.

Taking a moment to honour yourself for making time for your meditation practice – to connect with yourself with kindness.

Let’s begin by take three gentle and generous breaths into the body.

Inhaling through the nose and exhaling out through the mouth.

**breath in and out three times**

Allowing your breath to return to normal and noticing where you feel the breath the most – perhaps at the tip of the nostrils, the chest or belly. No-one spot is better than another.

Where do you feel it the most?

Continuing to breath naturally now, allowing the body to breath all on its own.

**pause**

As you continue to breath gently, bringing awareness to your body.

Noticing the contact you’re making with the floor or chair, the weight of gravity falling through you. Sensing the touch of your clothes or air on the skin.

Getting in touch with your body and noticing any sensations that you feel right now, perhaps tingling, throbbing or a sense of spaciousness, a sense of heaviness or lightness.

Whether those feelings are pleasant, unpleasant or neutral.

Recognising and accepting them and yourself just as you are. You don’t need to be any other way.

It might be really easy to focus your attention on the body and breath, it may be challenging, and that’s okay too. It’s natural for the mind to wander. It’s what the mind does. When you’ve become aware the mind is wandering, acknowledge yourself for noticing and kindly bring your attention back to the breath, without judgement.

Continue to tenderly breath into your body.

**pause**

Acknowledging any tension or stress in the body, recognising and allowing the feelings to be there. Being kind to yourself and acknowledging how human it is to feel discomfort and strain sometimes. Gently soothing this part of your body with your breath and allow it to soften any amount possible with your gentle awareness.

**pause**

Perhaps you notice emotions arising, gently acknowledge and lovingly breath into where you are noticing this emotion in the body. Releasing any expectation, as best as you can, that it should be any other way and offer yourself the space you need to just be right now. Just as a best friend would offer you a warm loving embrace, allowing your own attention to do the same right now.

Acknowledging that we are imperfect and whole beings and accepting that with an open heart.

Sometimes it helps us stay more present and gentle with ourselves, when we place our hands on our belly or heart and express kindness toward ourselves. Gently whispering these phrases to yourself.

- May I give myself the compassion that I need.
- May I learn to accept myself as I am.
- May I forgive myself.
- May I be strong.
- May I be patient.
- May I be kind to myself.

Repeating the phrase or phrases that resonate with you.

Noticing how it feels to receive your own kindness.

Allow your breath to be infused with affection for yourself and others. It may not come naturally right now, but setting the intention to cultivate affection and kindness to yourself and others. Acknowledging we all have moments of difficulty, pain and sorrow, as well as joy and happiness throughout our lives.

Breathing gently and affectively into your body for three more breaths being present to each one.

**ring bell**
